# The Argus-II Retinal Prosthesis Implantation; From the Global to Local Successful Experience

**DOI:** 10.3389/fnins.2018.00584

**Published:** 2018-09-05

**Authors:** Mohsen Farvardin, Mehrdad Afarid, Adel Attarzadeh, Mohammad K. Johari, Morsal Mehryar, M. Hossein Nowroozzadeh, Feisal Rahat, Hossein Peyvandi, Reza Farvardin, Mohammad Nami

**Affiliations:** ^1^Department of Ophthalmology, Poostchi Ophthalmology Research Center, School of Medicine, Shiraz University of Medical Sciences, Shiraz, Iran; ^2^Farvardin Eye Clinic, Shiraz, Iran; ^3^Computer Engineering Department, Sharif ICT Innovation Center, Sharif University of Technology, Tehran, Iran; ^4^Students' Research Committee, Shiraz University of Medical Sciences, Shiraz, Iran; ^5^Department of Neuroscience, School of Advanced Medical Sciences and Technologies, Shiraz University of Medical Sciences, Shiraz, Iran; ^6^DANA Brain Health Institute, Iranian Neuroscience Society, Fars Chapter, Shiraz, Iran

**Keywords:** argus II, artificial vision, retinitis pigmentosa, visual prosthesis, visual rehabilitation, programming, safety, outcome assessment

## Abstract

Over the past few years, visual prostheses (namely, Argus II retinal implant) and gene therapy have obtained FDA approval in treating blindness resulting from retinitis pigmentosa. Compared to gene therapy; Argus II is less costly with a demonstrated favorable outcome, though the vision is yet artificial. To obtain better results, expectation counseling and preoperative retinal assessment are critical. The global experience with Argus II has enrolled no more than 300 cases so far. The first Argus II retinal prosthesis in Iran was successfully implanted in Shiraz (October 2017). To date, Argus II artificial retina is implanted in four patients in Iran. Beside successful surgery and post-operative care, rehabilitation efforts with validated outcome measures including visual rehabilitation together with neurovisual, visuo-constructive and cognitive rehabilitation/empowerment approaches are expected to boost the functional outcome. A multidisciplinary approach within a cross-functional team would optimize strategies toward better patient outcomes. As such, establishing a collaborative network will foster organized research efforts to better define outcome assessment and rehabilitation strategies. This technology report paper has been an attempt to provide an overview of Argus-II retinal implant global experience as well as the clinical outcome of the so far cases in Iran. Insights from this report were communicated during the first “Brain Engineering and Computational Neuroscience Conference,” 31 January-2 February 2018 in Tehran.

## Background

The inherited retinal disease known as Retinitis Pigmentosa (RP) has an estimated prevalence of 1 in 4000 worldwide (Hartong et al., 2006). RP leads to degeneration of the photoreceptor layer of the retina, and while the condition is assumed to be linked to over 200 RP-causing mutations, they occur in almost 25 genes and a count of over 120 loci (Sohocki et al., 2001; Hagiwara et al., 2011). The residual inner retinal cells have prompted efforts to develop retinal prostheses to stimulate the surviving neural retinal cells and possibly restore functional vision.

Devices used to restore vision loss have long been ideas for science fictions. The captivating wish that technology may someday allow us to excel our physical limitations has long been with scientists, doctors and the public at large. The development of artificial vision began with occipital cortex prosthesis, though the retinal prosthesis has advanced faster over recent years (Fernandes et al., 2012). In reality, efforts to provide RP patients with artificial vision have resulted in the development of the Argus II Retinal Prosthesis System (Second Sight Medical Products, Inc., Sylmar, CA, USA) which acquired approvals by the European Union and the US Food and Drug Administration (FDA) in 2011 and 2013, respectively (Ghodasra et al., 2016).

In addition to Argus II retinal implant, the IRIS 2 (Pixium) and Alpha-AMS (Retina Implant) devices have received the EU CE mark (Hornig et al., 2017; Daschner et al., 2018).

Today, a considerable number of patients are using Argus II retinal prosthesis to regain some functional vision assisting them to make their way in the world. The system is a surgically implantable 60- electrode array and a receiver coil using an external video-processing unit to convert optical data captured from an eye-glass mounted video camera into electrical signals. The resultant evoked action potentials propagate to the visual brain via the optic nerve producing visual percepts (Zhou et al., 2013; Zrenner, 2013; da Cruz et al., 2016).

Since receiving approvals, the device has been used in almost 300 cases globally. The first Argus-II retinal prosthesis implantation in Iran was successfully done in October 2017 in Shiraz. So far, the four cases who underwent this procedure in our setting have demonstrated promising visual function results and improved performance on orientation and mobility tasks comparable to what has already been reported in the literature (Rizzo et al., 2014; Stronks and Dagnelie, 2014). Long-term surveillance studies have demonstrated the sustainability of outcome as well as safety and tolerability in up to 5 years of clinical follow-up (da Cruz et al., 2016).

Post-mortem eye investigations done in RP patients who were concurrent with the engineering of first-generation Argus system (Argus-I) confirmed that almost 80% of the inner nuclear layer and 30% of the ganglion cell layer in the maculae might survive (Baumgartner, 2000; Hamel, 2006).

The Argus II retinal prosthesis system surrogates the function of damaged photoreceptors and degenerated outer retinal cells through which the patient may regain functional visual abilities (Ghodasra et al., 2016).

Collective interdisciplinary experience needs to converge to optimize patient outcomes with the Argus II implants. To do so, potential challenges in preoperative screening, proper case selection, post-operative care, strategies for neurovisual and cognitive rehabilitation with measurable performance outcome through novel neurotechnological approaches need to be considered within retinal prosthesis cross-functional teams (Ghodasra et al., 2016).

Currently, there are two ongoing clinical trials i.e., the “Argus II Retinal Prosthesis System - Better Vision RP Study” and “Argus® II Retinal Stimulation System Feasibility Protocol.” These clinical trials are intended to investigate the clinical outcome measures following the implantation of the Argus II Retinal Prosthesis in patients with advanced RP who have a measurable central residual visual field smaller than or equal to 5° radius. The array is placed parafoveally, adjacent to the preserved central visual field (i.e., “tunnel vision”) in these subjects. These two studies address some key clinical outcome measures including adverse events, visual field, visual acuity, safety, activities of daily living, quality of life, orientation and mobility, spatial vision, stability of implant, and system functionality in a 2-year (clinicaltrials.gov number NCT03418116) and 5-year (clinicaltrials.gov number NCT00407602) time- frames.

The continued passionate hard work of researchers in ophthalmology, vitreoretinal surgery, visual science, cognitive neuroscience, neuroengineering and allied medical and engineering sciences is crucial to make this even more successful. Balancing the above with the spirit of patients willing to undergo the procedure and adhere to rehabilitation programs, gives hope to everyone with a stake in the enterprise that 1 day functional vision in RP may impartially be restored.

The significance of clinical outcome measures and lacking available guidelines for the rehabilitation process following artificial retinal implantation necessitate reviews on the topic. Moreover, the pros and cons of the related attributes upon case selection and screening, surgical procedure, safety, performance, outcome and utility, device programing as well as neuro-visual rehabilitation processes are yet to be widely discussed.

The present report is an attempt to provide an overview of the elements of success in Argus-II retinal implant at global level, and also to highlight the existing local experience with some remarks on the clinical outcome of the patients who have already undergone the process in Iran.

## Methods

Following a literature search using the combination of keywords Retinitis pigmentosa with Argus-II retinal prosthesis, neurovisual rehabilitation, programming, safety and functional outcome; an interdisciplinary team reviewed the available evidence. Our search in PubMed, MEDLINE, Scopus, Google Scholar and CINAHL databases yielded a total of 64 documents (April 2009-February 2018). The search strategy in the present report was more toward the devices that have been approved for clinical use mainly the Argus-II retinal implant system.

From the retrieved documents, 31 more relevant papers were isolated and circulated within the panel. Following a thorough review and plenary discussions, a summary report was communicated during the first “Brain Engineering and Computational Neuroscience Conference,” 31 January-2 February 2018 in Tehran. The objective of the conference was to converge collective experience and multidisciplinary insights in the field of brain engineering and computational neuroscience where the topic “neuro-prostheses” was the focus in some key-note talks. This manuscript discusses: 1- Argus-II retinal prostheses description, 2- case selection and screening, 3- surgical procedure, 4- safety, performance outcome and utility, 5-device programming and 6- neurovisual and cognitive rehabilitation, to highlight recommendations toward optimizing patient outcomes with the Argus-II system.

## Argus-II retinal prostheses description

This system which is also known as the bionic eye or the retinal implant works by stimulating the inner retinal neurons that survive retinal degeneration. An implanted patient would gain functional vision with closed eyes or opaque ocular media and their in-space navigation would need head-rotation instead of eye movements.

**Figure 1:**
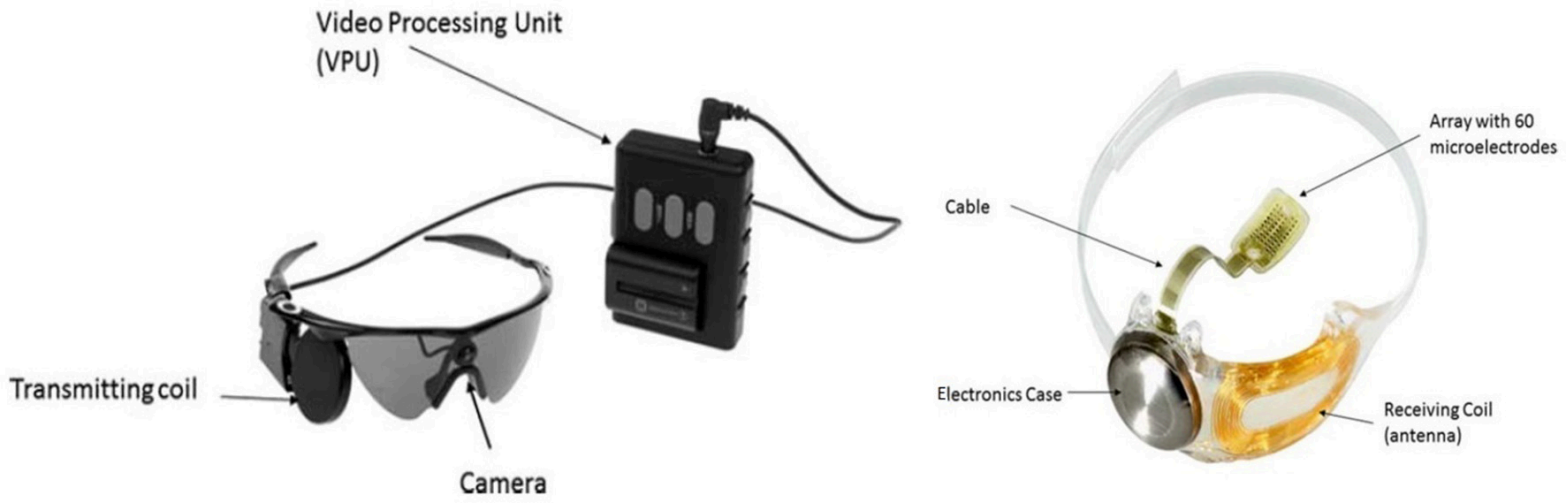
The Argus II Retinal Prosthesis System consists of implanted and external components. The implant is an epiretinal prosthesis that includes a receiver, electronics, and an electrode array that are surgically implanted in and around the eye. The array has 60 electrodes arranged in a rectangular grid, of which 55 are enabled. It is attached to the retina over the macula with a retinal tack. The external equipment includes glasses, a video processing unit (VPU) and a cable. The glasses include a miniature video camera, which captures video images, and a coil that transmits data and stimulation command to the implant. The VPU converts the video images into stimulation commands and is body-worn. The cable connects the glasses to the VPU. The Argus II System operates by converting video images into electrical energy that activates retinal cells, delivering the signal through the optic nerve to the brain where it is perceived as light. The Argus II Clinician Fitting System (CFS) and Psychophysical Test System (PTS) are used in the clinic to test and program the Argus II Implant and external equipment. Figure and description adapted with permission from Second Sight Inc. Data on File, April 2018.

The system possesses implanted and external components. The external part comprises a small CMOS camera mounted on a pair of glasses. The camera is cable-connected to the Video Processing Unit (VPU) worn on a belt. When the system is turned on, visual information is captured and translated into a real-time brightness map via the VPU. The brightness map data would get transferred through a radiofrequency (RF) link from the glasses-mounted external coil to the internal receiving coil already secured to the eye during the surgery (Zhou et al., 2013; Figure 1).

In Argus-II system, an inbuilt Application-Specific Integrated Circuit (ASIC) produces stimulus-adjusted pulses relayed to a 60-channel microelectrode epi-retinal array through a connecting cable. At retinal level, while the array rests in contact with the retinal surface over the macula, it is tacked to the retina with a metallic tack to allow the transmission of electronic signals received from the external part (Zhou et al., 2013).

Just recently, some image-processing software modifications have been applied to improve the Argus-II vision regarding edge-detection in grating visual acuity (GVA) leading to better shape and object recognition (Duncan et al., 2017).

Argus II developers have started to work on the next-generation implant with 240 electrodes, which can also be improved by peripheral electrodes. Their eventual goal is to minimize the size of the electrode similar to the retinal ganglion cell bodies, with the capacity of individual cell activation (Duncan et al., 2017).

## Case selection and screening

Having been approved for RP patients so far, the Argus II System is indicated for use in adults aging 25 years or older who have severe to profound outer retinal degeneration while possessing some residual light perception. They should also have had the previous history of useful form vision to guarantee the proper cortical response. In case no residual light perception remains, the retina should be responding to electrical stimulation before patients are selected for the intervention (Dagnelie et al., 2017).

Contraindications include: 1- ocular diseases or conditions that could prevent the Argus II System from working (e.g., optic nerve disease, central retinal artery or vein occlusion, history of retinal detachment, trauma, severe strabismus), 2- ocular structures or conditions that could prevent the successful implantation of the Argus II Implant or adequate healing from surgery (e.g., extremely thin conjunctiva, axial length <20.5 or >26 mm, corneal ulcers, choroidal neovascularization in the area of the intended tack location, etc.), 3- ocular diseases or conditions (other than cataracts) that prevent adequate visualization of the inner structures of the eye (e.g., corneal opacity, etc.), 4- inability to tolerate general anesthesia or the recommended antibiotic and steroid regimen associated with the implantation surgery, and 5- predisposition to eye rubbing (Zhou et al., 2013; Stronks and Dagnelie, 2014; Ghodasra et al., 2016), and 6- lack of communicative capacity in order to work with the care providers and optimally use the VPU (including intellectual challenge, Usher syndrome, deafness, etc.) (Ghodasra et al., 2016).

Patients should be allowed to examine and wear the external equipment before the final decision. Counseling patient expectations has been recognized as a critical constituent of the patient selection process (Teutsch, 2003). Patients need to be advised that the output from the device would be a whole new type of functional vision rather than restoration of previous vision. This holds an even more significance since the implantation of artificial retina requires a considerable investment. Although a study confirmed that Argus II implantation vs. usual care in RP is a cost-effective intervention, the high initial costs of the procedure should always be considered upon clinical decisions (Vaidya et al., 2014). Over and above, patients' compliance with frequent follow-up assessments and the rehabilitation program should be taken into account (Ahuja and Behrend, 2013).

Characteristics such as realistic expectations, supportive family, existing blindness skills such as familiarity with available devices for low vision and blindness, baseline functional status of the patient including general health, cognition, and communication are known to be positively correlated with more favorable outcomes (Ahuja and Behrend, 2013; Chuang et al., 2014).

A low-vision specialist and non-physician support staff should begin the screening process for Argus-II. Patients with relatively good vision and those whose diagnosis fall outside RP should be initially excluded. A full ophthalmology examination including anatomical and functional assessments should allow determining factors leading to successful implantation. The anterior segment needs to get examined for conjunctival or scleral thinning and the lens status. An indirect funduscopic examination should also be done to document the possible presence of macular scar, posterior staphyloma, epiretinal membrane, retinal tear, optic disc cupping and to evaluate posterior vitreous detachment (PVD) status (Chuang et al., 2014; Olmos de Koo and Gregori, 2016).

Other preoperative assessments include but not restricted to wide-field fundus photography, Optical Coherence Tomography (OCT), ocular ultrasonography (A-scan, B–scan), flash Visual Evoked Potentials (fVEP), neurofunctional and visuocortical assessments such as functional and structural neuroimaging and brain-mapping as well as review of consent forms, systemic evaluation for general anesthesia, introducing post-operative rehabilitation roadmap, and psychological counseling to manage expectations (Castaldi et al., 2016; Ghodasra et al., 2016).

In addition, factors including motivation and neurocognitive ability, desire to improve skills, willingness to accept instruction, ability to devise and implement strategies for new tasks, ability to set goals and work toward them would be expected to result in better outcomes (Ahuja and Behrend, 2013; da Cruz et al., 2013).

## Surgical procedure

As a new era in vitreoretinal surgery, implantation of Argus-II would put forward set of surgical challenges. The advent of “silico-biologic” interface refines a variety of techniques already been known to vitreoretinal surgeons. In phakic patients, phacoemulsification has to be done prior to Argus II implantation (Luo and da Cruz, 2016).

Based on the existing literature, after a 360° limbal conjunctival peritomy is performed, the receiving coil is inserted under the lateral rectus muscle and extended into the infratemporal quadrant; the electronics case is placed on the supratemporal sclera and then according to axial length-related tables sutured to the sclera through tabs located on the band. Then, a 3-port complete pars plana vitrectomy including the detachment of the posterior vitreous and meticulous removal of the peripheral vitreous is performed. Through a 5.2 mm supratemporal sclerotomy (which is created with a special knife), the electrode array is introduced into the eye and subsequently secured to the retina-choroid-sclera with a custom-made titanium retinal tack. The sclerotomy is then sealed with sutures, and all other sclerotomies are closed. Surgical time generally falls between one and a half and 4 h (Ghodasra et al., 2016; Luo and da Cruz, 2016).

The system configuration followed in the operation room includes configuring the device components including the Clinician Fitting System (CFS), Communication Adapter (CA) and Operating Room Coil (inserted into the sterile sleeve) (Olmos de Koo and Gregori, 2016).

**Figure 2:**
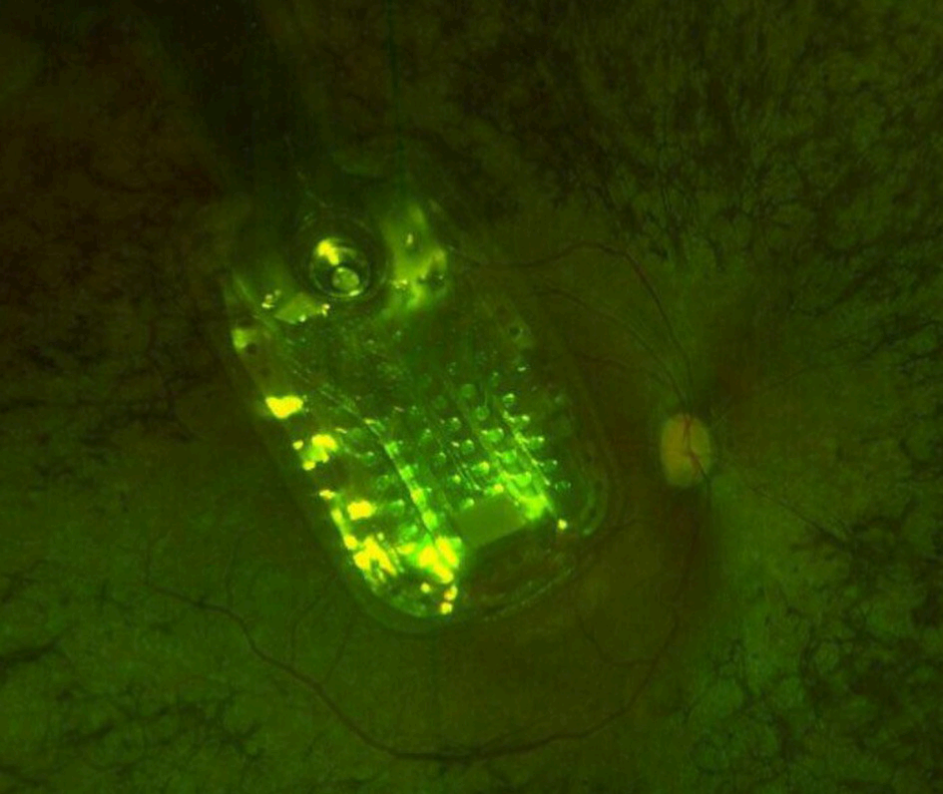
Placement of the Argus-II micro-electrode in our first implanted patient. Image from Farvardin Eye Clinic 2017, Shiraz, Iran.

Handling the delicate electronics which are done with silicone tipped forceps should be done carefully during the operation. Postoperatively, patients are given steroid and antibiotic eye drops and are advised to refer for typical postoperative follow-up visits for monitoring possible adverse events (Ghodasra et al., 2016; Luo and da Cruz, 2016; Olmos de Koo and Gregori, 2016). Figure 2 demonstrates the placement of Argus-II micro-electrode array in our first implanted patient.

## Safety, performance outcome and utility

A 3-year surveillance study on Argus II by Humayun et al. has shown some surgery-related adverse events such as endophthalmitis, hypotony, and conjunctival erosion or dehiscence. Patients performed significantly better with the Argus II on than off on all visual function tests and functional vision tasks (Humayun et al., 2012). Twenty-four of 30 patients remained implanted with functioning Argus II Systems at 5 years after implantation. The procedural and device refinement has further improved the adverse-event profile since then (da Cruz et al., 2016).

The hypotony occurs after Argus II implantation usually relates to the inadequate closure of sclerotomies. In such an instance, close follow-up with pressure patching might be all needed in case no other serious adverse signs are present (Ghodasra et al., 2016).

Several techniques have also been proposed to prevent conjunctival erosion. For example, the array cable and the anterior edge of the coil need to be covered with processed pericardium or donor corneal graft. Tenon's membrane would also need closure before conjunctival closure. In addition, Nylon sutures are preferred since the braided nature of Mersilene polyester sutures may contribute to erosion. Knots need to be rotated posteriorly beneath suture tabs to decrease the risk of eroding conjunctiva. In case conjunctival erosion is confirmed post-operatively, the patient would receive topical antibiotics and return to the operating room for closure (Ghodasra et al., 2016).

Given the adherent nature of vitreous cortex in patients with RP, excessive traction on the retina should be avoided. Moreover, peeling of the macular epiretinal membranes would help efficient contact between the electrodes and retina. Nevertheless, the internal limiting membrane should remain unpeeled to avoid the possibility of retinal holes in the macula. Once localized retinal detachments or teats are observed post operatively, laser retinopexy needs to be pursued away from the array (Hamel, 2006; Zhou et al., 2013). Other than the above, a rare but potential complication i.e., infectious endophthalmitis should always be considered in Argus II implanted patients. Based on the available reports on procedural safety and post Argus-II implantation safety follow-ups to date, all cases of endophthalmitis in Argus II patients have successfully been resolved with no device explanted following early diagnosis and proper treatments (da Cruz et al., 2016).

Based on the above, it appears that patients should be trained about potential signs and symptoms of postoperative complications such as endophthalmitis and conjunctival erosion, since timely detection of such complications or challenges is the key to maintaining successful outcomes (Ahuja and Behrend, 2013).

Concerning the outcome measures in motion test, so far reports have shown that Argus-II patients perform better with the system ON vs. system OFF over the study course (Ghodasra et al., 2016). On the Grating Visual Acuity (GVA) test, up to one-third of subjects were able to reliably score 1.6 and 2.9 LogMAR at least once with the system ON, whereas no Argus-II patient could score on the scale with the system OFF (Dagnelie et al., 2017).

The Argus II system ON vs. OFF could also provide patients with significant improvement in orientation and mobility tests i.e., finding a door and following a line. These tests are referred to as a “door task” and “line task” when assessing functional vision of patients with ultra-low vision (Ghodasra et al., 2016; Duncan et al., 2017).

Self-report questionnaires including Massof Activity Inventory and VisQOL informed mild improvement in activities of daily living and quality of life following the use of Argus-II in RP patients (Singer et al., 2012; Duncan et al., 2017).

There have been some custom end-point measures to assess functional outcomes of the Argus-II clinical trial patients. Methods to assess visual performance in such patients comprise grating visual acuity, square localization, and direction of motion (Dagnelie et al., 2017). In addition, studies have developed the Functional Low-Vision Observer Rated Assessment (FLORA) due to the lack of qualified outcome measures when evaluating impacts on quality of life. The test is however complex and its subjectively reported measures are hard to quantify (Baumgartner, 2000). Some adaptive versions of currently employed tools have been developed and are being further refined (Dagnelie et al., 2017).

## Device programing

Device programing is pursued after the Argus II device is implanted and fitted, and before the camera can be turned on. The first session would examine which electrode on the array is functionally usable, and which electrode yields a too high resistance value. A quick array scan at different stimulation amplitudes would be then performed to distinguish the array electrodes which reliably yield percepts, or produce phosphenes. Later, during the second session, the minimum current needed to exert a percept which the patient can see half of the time will be defined for the electrodes which yielded a percept during array scanning. A map through which the video signal from the camera transposes to the electrical signal for individual or groups of electrodes, i.e., the Video Configuration File (VCF) is then generated. This file determines both the frequency and number of electrodes simultaneously stimulated. A set of various VCF configurations and image processing filters are saved onto the patient's VPU to be applied in different conditions such as normal light conditions, contrast enhancement for low light conditions, and a setting for edge detection.

The final programming phase before the camera is turned on would compensate the angle at which the array was placed on the retina during surgery (Luo and da Cruz, 2016).

Some new programming software tools are designed to simplify the programming process. This new software named Programming Assistant is being tested in a small clinical trial, and approval for its launch will be sought provided the patients' performance is not negatively impacted. Some modifications in glasses design have also been underway to improve users' comfort (Ghodasra et al., 2016; Dagnelie et al., 2017).

## Neurovisual, visuoconstructive, and cognitive rehabilitation

Despite all the achievements, today's retinal prostheses provide limited vision. Though some individuals perform quite well in object and shape recognition tasks (da Cruz et al., 2013), most Argus II patients report visual percepts best described as moving shadows (Dagnelie et al., 2017).

Secondary to challenges in quantifying the visual improvement in Argus-II patients, regulatory bodies put forward issues against market approvals. For example in the US, letter visual acuity, contrast sensitivity, visual field testing are the least standardized measures to define functional vision while almost no Argus II user has such measurable improvements (Dagnelie et al., 2017). Meanwhile, the visual benefits of Argus-II become more tangible when compared with patients with ultra-low vision i.e., hand motion, light projection or light perception (Humayun et al., 2012; Dagnelie et al., 2014; Stronks and Dagnelie, 2014).

Some testing methods used with Argus II both during the feasibility and the post-approval studies have aimed at target localization, motion direction discrimination and GVA (Dorn et al., 2013). Investigators have demonstrated a notably improved capacity in identifying high-contrast shapes and objects in a good fraction of implanted patients (Ghodasra et al., 2016; Dagnelie et al., 2017).

Likewise, studies showed a considerable improvement in target localization and motion direction discrimination (89 and 56%, respectively) in Argus-II patients in system ON vs. OFF examinations (Ghodasra et al., 2016; Luo and da Cruz, 2016; Duncan et al., 2017).

The GVA, which was originally developed as a research paradigm, tests the individuals' ability to differentiate the orientation of black and white gratings at different spatial frequencies (Dorn et al., 2013; Ghodasra et al., 2016). In a recent study, while no participant could demonstrate any measurable capacity with the system OFF, almost a half and one-third of 1- and 3-year post-implantation subjects, respectively, scored 2.9 LogMAR or more with the system ON (Dagnelie et al., 2017).

The Visual Rehabilitation Program is applied to all Argus II patients who have undergone customization and training in the clinic. A Rehabilitation Kit containing objects such as lights and high-contrast shapes for use during the sessions is be provided. Rehabilitation sessions are scheduled on case by case basis while all patients who use the device need to refer to the clinic each month and follow their home assignments. The goal of the visual rehabilitation process is to help the patients maximize the use of the visual information in order to improve their quality of life.

When it comes to the visual brain a set of complex pathways should be considered. Those range from retinofugal fibers to multiple subcortical structures involved and ultimately the visual cortex which serve neurocognitive processing together with remote cortical regions of the brain (Grossberg et al., 1997). Hypothetically, reanimation of the retina would provide the visual cortex with continued impulses with the device ON, and this would augment the visual cortex and associated pathways through neural plasticity (Lambert et al., 2004).

This can prompt researchers to design and pursue preoperative and post-operative neurovisual, visuoconstructive (coordination of fine motor skills with spatial abilities, usually in the reproduction of geometric figures) and cognitive assessments which not only provide insights into the possible structural and functional benefits gained following Argus-II implantation, but also help to better strategize visual and neurocognitive rehabilitation.

Rehabilitation aims to facilitate the integration of engineers new visual inputs and the earlier vision possessed in order to enhance patients' quality of life and independence. The rehabilitation process includes in-clinic as well as community settings (Ghodasra et al., 2016; Olmos de Koo and Gregori, 2016; Dagnelie et al., 2017).

**Figure 3:**
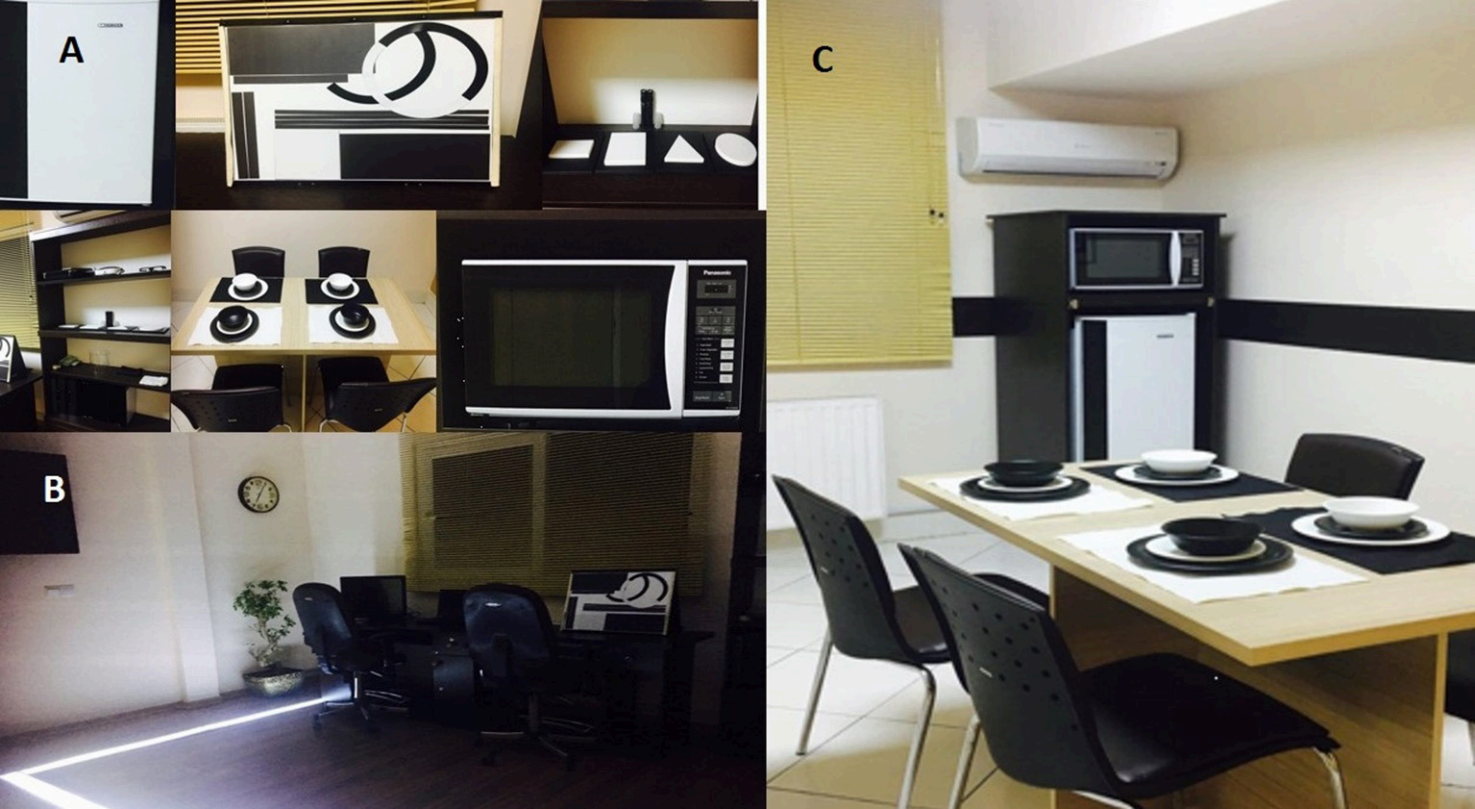
Visual and functional rehabilitation platforms including a set of equipment as per the guidelines and recommendations laid down by the Argus II system developer though which serial rehabilitation sessions would be completed to improve functional vision. The visual rehabilitation process is followed intermittently in visual rehabilitation unit and functional rehabilitation set up. Things to consider include contrast modifications, lighting evaluation and contrast evaluation when trainings are provided. It is also recommended to similarly customize the environment of the patients' home. **(A)** The functional rehabilitation unit where subjects are trained to undertake target localization tasks on a magnet board, touch items, focus of contrasts and edges detection with the system ON, and to interact with the customized environment by locating and working with the microwave oven, fridge, locating black and white bowls, plates and table cloth, etc. **(B)** The visual rehabilitation setup comprising the grating visual acuity task on computer monitors, visual rehabilitation kit provided by the developer and the line-task in which the subject needs to track the path of light-emitting target on the floor. **(C)** Another view from the functional rehabilitation unit designed for subjects using the Argus II device. Images from Farvardin Eye Clinic, Shiraz, Iran.

The Instructional kit provided by the developer (www.secondsight.com) as a collection of high contrast items, such as white shapes against a black background, as well as black plates and white bowls. Like other investigators, our team uses the same set of instruments in two laboratories including the visual rehabilitation and visuofunctional platforms (Figure 3). In addition, the neuroscience laboratory (Brain, Cognition, and Behavior) in our setting provides cognitive, electrophysiology and imaging facilities to comprehensively assess candidates also from the visual neuroscience perspective. All selected candidates for Argus II implantation would preoperatively undergo comprehensive psychological evaluations, a 32-chanel quantitative electroencephalography (qEEG) with photic stimulation brain mapping for cortical excitability assessment, conventional MRI and related cortical volumetric and morphometric assessments as well as 12-channel optical neuroimaging using our functional near infra-red spectroscopy (fNIRS) setup. Same set of evaluations are pursued over the follow-up time points during the neurovisual rehabilitation process.

When it comes to continued neurovisual rehabilitation with the device ON, some key issues including patient fatigue and oversaturation need to be taken into account (Ghodasra et al., 2016). Additionally, new visual inputs from the Argus II system may not be easy to interpret and adaptation to the electrical stimulation may occur and percepts can get dimmer after an extended period of device use (Dagnelie et al., 2017).

Other than teaching patients how to optimally integrate the new visual inputs into their daily life, rehabilitation experts need to manage the expectations of patients and their families as to the degree of vision the patient will experience (Dagnelie et al., 2014).

## Our promising local experience with the Argus-II retinal implant in iran

In our setting, Argus-II retinal prostheses have so far been successfully implanted in 4 patients. In the fitting process, 60/60 of the electrodes were activated in all cases with low impedance (less than 50 kΩ). All 4 patients were able to perceive hand motion and vague pattern recognition following the fitting procedure.

After two visual rehabilitation sessions, all patients had measurable grating visual acuity from 2.6 to 2.9 LogMAR (2.72 ± 0.24).

In addition, the results of visual evoked potential (VEP) tests showed improvement when devices were switched on compared to the switched-off state.

With regard to surgical complications, one patient underwent successful re-tacking right after the first tacking due to inappropriate electrode array position. One patient had moderate vitreous cavity hemorrhage which was cleared after 3 weeks without sequela.

No patient developed endophthalmitis, persistent inflammation, rise in the intra-ocular pressure, or exposure of the implant.

## Concluding remarks

The working-team concept is crucial to success in many multidisciplinary medical projects and Retinal Prosthesis team is perhaps a typical example. Synergizing efforts made by vitreo-retinal surgeons, medical engineers, rehabilitation experts, clinical and cognitive neuroscientists, and industry representatives would help moving toward more promising results.

Practitioners need to ensure that the patient selection for device implantation fulfills the eligibility criteria including patient's motivations, expectations, cognitive and communication capabilities as well as physical abilities to receive benefit from the device.

Key potentials for continued research toward improving the Argus-II vision through device optimization and advanced programing as well as neurovisual, visuoconstructive and cognitive rehabilitation make the present time a critical turning-point for retinal prosthetic systems such as artificial retina to drive even-better future outcomes.

## Ethics statement

All implanted subjects we briefed about the operation procedure, device specifics, cost vs. utility, realistic expectation and possible risks, after which they signed an informed consent. There was a clear mention in their consent that their clinical results might be included in our research database. The consent form and ethical standards followed the guidelines laid down by the ethical committee at Shiraz University of medical Sciences.

## Author contributions

All authors contributed to data review and plenary discussion upon the preparation of this report. MA, AA, MJ, MM, MoN, and FR have made an equal contribution to this report and thus been sorted alphabetically, identically as second-order authors. MoN drafted the manuscript and coordinated the data review process amongst authors. All authors were involved in the preparation, review, and approval of the manuscript. The remaining listed authors provided the manuscript with intellectual contents.

### Conflict of interest statement

The authors declare that the research was conducted in the absence of any commercial or financial relationships that could be construed as a potential conflict of interest.
